# llperm: a permutation of regressor residuals test for microbiome data

**DOI:** 10.1186/s12859-022-05088-w

**Published:** 2022-12-12

**Authors:** Markus Viljanen, Hendriek Boshuizen

**Affiliations:** National Institute for Public Health and the Environment - RIVM, PO Box 1, 3720 BA Bilthoven, The Netherlands

**Keywords:** Microbiome, Bioinformatics, Statistics

## Abstract

**Background:**

Differential abundance testing is an important aspect of microbiome data analysis, where each taxa is fitted with a statistical test or a regression model. However, many models do not provide a good fit to real microbiome data. This has been shown to result in high false positive rates. Permutation tests are a good alternative, but a regression approach is desired for small data sets with many covariates, where stratification is not an option.

**Results:**

We implement an R package ‘llperm’ where the The Permutation of Regressor Residuals (PRR) test can be applied to any likelihood based model, not only generalized linear models. This enables distributions with zero-inflation and overdispersion, making the test suitable for count regression models popular in microbiome data analysis. Simulations based on a real data set show that the PRR-test approach is able to maintain the correct nominal false positive rate expected from the null hypothesis, while having equal or greater power to detect the true positives as models based on likelihood at a given false positive rate.

**Conclusions:**

Standard count regression models can have a shockingly high false positive rate in microbiome data sets. As they may lead to false conclusions, the guaranteed nominal false positive rate gained from the PRR-test can be viewed as a major benefit.

## Introduction

Statistical tools and computational methods are important in analysing microbiome data. Modern microbiome data sets are created by sequencing marker genes or the entire metagenome in a sample, and mapping these sequences to operational taxonomic units (OTUs), amplicon sequence variants (ASVs), or species or other phylogenetic levels [1]. We refer to these microbiome units as taxa regardless of the aggregation level. A data set typically has hundreds to thousands of taxa and comparatively few samples. The sample is described by sampling unit (e.g. subject) and environmental characteristics. These additional variables are important because the microbiome (unlike the genome) can both modify and be modified by these factors [2].

The goal of statistical analysis is to identify associations between the microbiome and biological, environmental, genetic, clinical or experimental conditions, while taking into account possible confounding factors [3]. The research hypothesis is typically formulated as a null hypothesis, such as “There is no difference in the microbiome composition of comparison groups”. Several different types of analyses can be considered. A common statistical analysis of microbiome data is differential abundance (DA) testing, where each taxon is sequentially tested for a difference in taxon abundance given the experimental groups and covariates in the sample [4].

Classic statistical tests, such as Pearson correlation, T-test and ANOVA, are used to compare groups in microbiome data [5–8] even though the distributional assumptions can be suspect. When there are covariates under consideration, standard regression approaches have become popular tools. The Negative Binomial distribution, and packages like edgeR and DESeq2 based on it, are sometimes recommended for microbiome data. [9, 10]. While simulation studies show good performance, it has been pointed out that more realistic data do not satisfy their distributional assumptions [2, 11]. This can result in many false positives, implying the methods have a poor False Positive Rate (FPR) control [2, 4, 9, 10, 12–15].

Permutation tests provide a robust non-parametric approach for a comparison of experimental groups because the FPR is maintained at the nominal level [16]. With a limited number of confounding factors, stratification can be employed [17]. However, if the data set has a small sample size and multiple covariates, a regression approach with similar robustness properties as a permutation test is desired. Permutation of Regressor Residuals Test (PRR-test) [18] method controls the FPR within the regression approach, enabling a robust test of comparison groups or environmental gradients while taking into account the covariates. An R package ‘glmperm’ [18] provides this for the Generalized Linear Model (GLM) family, which does not contain count regression with zero-inflation that is characteristic of microbiome data [19]. In this paper, we present an extended R package ‘llperm’ (Log-Likelihood) suitable for microbiome data, which implements popular overdispersed and zero-inflated count regression models in this framework.

## Methods

### Testing differential abundance

For person $i=1,\ldots , n$ and taxa $j=1,\ldots , m$, define the detected counts $Y_{i,j}$ as a matrix $Y\in {\textbf {N}}^{n \times m}$. Our goal is to detect the differentially abundant taxa, which we denote by the binary vector $y^{*}\in \{0,1\}^{m}$ where $y^{*}_{j} = \mathbb {I}(\text {taxa } j \text { is differentially abundant})$. The null hypothesis is that there is no difference in the counts of a taxa between the experimental groups. We test hundreds of taxon *j* and obtain a vector of *p* values $p_j\in [0,1]^{m}$ from a single experiment. A good statistical hypothesis test should have the ability to 1) control the probability of a type I error (false positive result) at the nominal significance level $\gamma$, and 2) have sufficient power (i.e. true positive rate) for detecting the differentially abundant taxa. [11]. We quantify the FPR and power of the test with:

$$\begin{aligned} \begin{array}{c} \text {FPR} = \frac{\sum _{j=1}^{m}\mathbb {I}(p_i< \gamma \text { and } y^{*}_{j} = 0)}{\sum _{j=1}^{m}\mathbb {I}(y^{*}_{j} = 0)} \\ \text {TPR} = \frac{\sum _{j=1}^{m}\mathbb {I}(p_i < \gamma \text { and } y^{*}_{j} = 1)}{\sum _{j=1}^{m}\mathbb {I}(y^{*}_{j} = 1)} \end{array} \end{aligned}$$

### Model definition

Given that microbiome data often contain many zero counts, we define both single distribution models as well as zero-inflated models, consisting of a part modelling the probability of a zero (‘zero’ component) and a part modelling the number of counts (the ‘count’ component). Define the ‘count’ component related covariates as a matrix $X\in {\textbf {R}}^{n \times (p+q)}$ with *p* columns related to the covariate of interest and *q* other columns. Define the ‘zero’ component related covariates as a matrix $Z\in {\textbf {R}}^{n \times (s+t)}$ with *s* columns related to the covariate of interest and *t* other columns. Define the corresponding coefficient vectors $\overline{\alpha }\in {\textbf {R}}^{p+q}$ and $\overline{\beta }\in {\textbf {R}}^{s+t}$. This generalizes the simple case $X = Z$ where same covariates are considered to influence both counts and zero-inflation, as well as the single distribution model where the ‘zero’ component is omitted. Define the likelihood function for taxa *j*:

$$\begin{aligned} L_{j}(Y, X, Z, \overline{\alpha }, \overline{\beta }) = \prod _{i=1}^{n} f(Y_{i,j}, X_{i,:}, Z_{i,:}, \overline{\alpha }, \overline{\beta }) \end{aligned}$$

Denote the maximum likelihood solution $\hat{\overline{\alpha }}, \hat{\overline{\beta }}$:

$$\begin{aligned} \hat{\overline{\alpha }}, \hat{\overline{\beta }}:= \text {argmax}_{\overline{\alpha }, \overline{\beta }} L_{j}(Y, X, Z, \overline{\alpha }, \overline{\beta }) \end{aligned}$$

We factorize the matrices *X* and *Z*, and the corresponding coefficients $\overline{\alpha }$ and $\overline{\beta }$, into covariates of interest $X^{*}\in {\textbf {R}}^{n \times p}, Z^{*}\in {\textbf {R}}^{n \times s}$ and other covariates $X^{\dagger }\in {\textbf {R}}^{n \times q}, Z^{\dagger }\in {\textbf {R}}^{n \times t}$:

$$\begin{aligned} \begin{array}{cc} X = (X^{*}, X^{\dagger }) &{} \overline{\alpha } = (\overline{\alpha }^{*}, \overline{\alpha }^{\dagger })\\ Z = (Z^{*}, Z^{\dagger }) &{} \overline{\beta } = (\overline{\beta }^{*}, \overline{\beta }^{\dagger }) \end{array} \end{aligned}$$

The null hypothesis is that the regression coefficients for the covariate of interest is zero for both components is $\overline{\alpha }^{*}=0$ and $\overline{\beta }^{*}=0$. It is also possible to test only one covariate of interest while taking into account the other in model fitting.

### Permutation scheme

After a likelihood model *f* has been specified, a *p* value is calculated using both a standard likehood-ratio test and a permutation of regression residuals test [18]. We explain how to calculate these in three stages:

#### Calculate residuals for the covariate of interest from a least squares problem

The basic idea of the PRR test is that we replace the covariate of interest by their residual given by a linear regression on the remaining covariates. We first predict the covariate of interest $X^{*}$ from the other covariates $X^{\dagger }$ by solving the least squares problem $\hat{\Sigma }:= \text {argmin}_{\Sigma } \Vert X^{*} - X^{\dagger }\Sigma \Vert ^{2}$, and then we calculate the residuals $\tilde{X}:= X^{*} - X^{\dagger }\hat{\Sigma }$. While $X^{*}$ may be correlated with $X^{\dagger }$, replacing it by the residual $\tilde{X}$ ensures that it is not correlated. The same is done with *Z* to obtain $\tilde{Z}$. The maximum value of the likelihood is the same with the residuals as it is with the covariates of interest. We then permute the residuals to estimate the null distribution and therefore the *p* value. In case $X^{*}$ (and $Z^{*}$ when present) is a categorical variable with m categories, it is represented in the model matrix as a set of m-1 dummy variables, and the least squares problem consists of a system of m-1 regression equations, delivering m-1 residuals, which are used in place of the dummy variables.

#### For each resampling iteration, calculate *p* values using the permuted residuals

For every resampling iteration $b=1,\ldots , B$, use $\mathcal {I}_{b}(n)$ to denote a random permutation of row indexes $\{1,\ldots ,n\}$. We then substitute the factorized matrices *X* and *Z* by matrices without/with the permuted residuals:

$$\begin{aligned} \begin{array}{cc} X^{0} = (\tilde{X}, X^{\dagger }) &{} X^{b} = (\tilde{X}_{\mathcal {I}_{b}(n),:}, X^{\dagger })\\ Z^{0} = (\tilde{Z}, Z^{\dagger }) &{} Z^{b} = (\tilde{Z}_{\mathcal {I}_{b}(n),:}, Z^{\dagger }) \end{array} \end{aligned}$$

The likelihood ratio pivotal has an asymptotic Chi-squared distribution, from which a *p* value can be calculated given the maximum likelihood solutions $\hat{\overline{\alpha }}^{0}, \hat{\overline{\beta }}^{0}$ and $\hat{\overline{\alpha }}^{b}, \hat{\overline{\beta }}^{b}$ of the unpermuted and permuted residuals in the matrices, respectively:

$$\begin{aligned} p_{j,b} = \mathcal {X}^{2}_{p+q}\left( -2 \text {ln}\left( \frac{L_{j}(Y, X^{b}, Z^{b}, \hat{\overline{\alpha }}^{b}, \hat{\overline{\beta }}^{b})}{L_{j}(Y, X^{0}, Z^{0}, \hat{\overline{\alpha }}^{0}, \hat{\overline{\beta }}^{0})}\right) \right) \end{aligned}$$

where p and q are the number of columns in $X^{*}$ and $Z^{*}$ respectively, that is 1 in case of a continuous variable, and m-1 in case of an variable with m categories.

#### Calculate a *p* value

First, a *p* value based on the standard likelihood-ratio test can be calculated:

$$\begin{aligned} \hat{p}_{j} = \mathcal {X}^{2}_{p+q}\left( -2 \text {ln}\left( \frac{L_{j}(Y, X^{\dagger }, Z^{\dagger }, \hat{\overline{\alpha }}^{\dagger }, \hat{\overline{\beta }}^{\dagger })}{L_{j}(Y, X^{0}, Z^{0}, \hat{\overline{\alpha }}^{0}, \hat{\overline{\beta }}^{0})}\right) \right) \end{aligned}$$

Second, a *p* value based on permutation of regression residuals can be calculated based on the resampling iterations:

$$\begin{aligned} p_{j} = \frac{1}{B}\sum _{b=1}^{B} \mathbb {I}(p_{b,j} < \hat{p}_{j}) \end{aligned}$$

### Model regression specification

The likelihood of a single observation $f(Y_{i,j}, X_{i,:}, Z_{j,:}, \overline{\alpha }, \overline{\beta })$ can have an arbitary specification in our model. We consider the following eight regression models in the experiments, which can be classified as Poisson or Binomial type models with zero-inflation and/or overdispersion (compound Gamma or Beta-distribution) illustrated in Tables 1 and 2.

**Table 1:** Poisson family of models

Zero-inflation	Overdispersion	
	No	Yes
No	Poisson	Negative binomial
Yes	ZI Poisson	ZI negative binomial

**Table 2:** Binomial family of models

Zero-inflation	Overdispersion	
	No	Yes
No	Binomial	Beta binomial
Yes	ZI binomial	ZI beta binomial

The raw abundance counts are not directly comparable across samples in real data sets. These counts do not directly reflect the true amount of DNA, but also sample DNA quality, concentration, amplification, barcoding and sequencing act as factors which make the taxa counts in a sample larger or smaller in an unpredictable way [20, 21]. Therefore the taxon abundance can only be analysed relative to the library sizes $s_{i} = \sum _{j=1}^{m} Y_{i,j}$ [4, 11]. This is directly incorporated in the Binomial distributions because the counts are drawn from the library size. Poisson distributions can include an $\text {offset}(\text {log}(s_{i}))$ term in the regression equation to include the library size.

### Model implementation (llperm)

We propose an R package called ‘llperm’ that implements the model. Our package extends the ‘glmperm’ R package implemented by Werft [18], which in turns is an extension of ‘logregperm’ R package proposed by Potter [22]. The original package implemented the novel permutation test procedure for inference in logistic regression models, whereas the glmperm extended this into Generalized Linear Models (GLM) where more than one covariate can be involved together with the covariate of interest. Our package in turn extends this implementation in three ways to better fit microbiome data:

1. The covariate of interest can occur as a category with multiple levels.
2. We generalized the implementation to any likelihood based model, which enables additional distributions with zero-inflation and overdispersion (Poisson, ZIPoisson, NegBin, ZINegBin, Bin, ZIBin, BetaBin, ZIBetaBin,...).
3. In case of zero-inflated models, the regression coefficients related to the count- and the zero-component can be simultaneously tested.

See the “Appendix” for a simple example using the R formula syntax.

## Simulations

We performed simulations in order to validate our method. When validating a method, it is important that the simulations resemble real life situations, and not an artificial situation in which the assumptions of the method are met. Therefore we use the real dataset as the foundation for generating simulated data where the ‘signal’, i.e. truly differentially abundant taxa, is known.

### Real data underlying the simulation

**Fig. 1:**
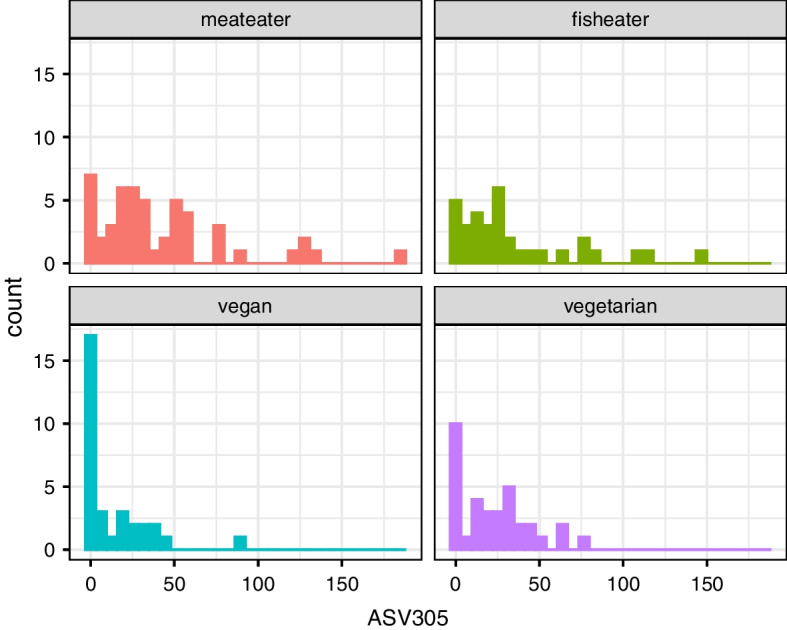
Example of raw data: 16S rRNA sequence counts for a single taxa ‘ASV305’, which appear overdispersed (mean 30, variance 1133) and zero-inflated, possibly influenced by Diet

The VEGA data set [23] studied the extent to which antibiotic resistant bacteria occur in vegetarians and non-vegetarians. Faecal samples were collected from volunteers and used to detect the Extended-Spectrum Beta-Lactamases (ESBL) producing bacteria, while 16S rRNA sequencing was used to see what microbiota were present. These data can also be used to study the relation between microbiota abundance and diet (vegan, meateater, fisheater, vegetarian), taking into account confounders such as sex, age, urbanization, pets at home, medication and travel history. The data set has 149 persons and 531 ASVs that occur in at least 10% of persons. The microbiome is therefore represented by a 149 × 531 table of counts. For example, the counts for ‘ASV305’ in Fig. 1 could indicate some difference in diet groups.

### Simulated data

#### Adding signal to the real data

For each simulated dataset, we assigned each person in our data to one of 4 groups (meateater, fisheater, vegetarian, vegan) with equal 25% probability, irrespective of his/her real status. In each group, 10% of the taxa are randomly chosen to be differentially abundant. If a taxa is differentially abundant in a person, the counts are multiplied by an effect size (+25%, +50%, +100%, +200%, +400%) [4, 9, 24, 25]. However, note that this only modifies non-zero counts.

We additionally introduced signal in the zero counts by decreasing their probability. For every taxon, we first calculated the baseline odds of the counts being non-zero, and assigned this to every individual. If the taxon is differentially abundant in a given person, this odds was multiplied by the effect size, and the probability of a non-zero sample was calculated from this increased odds. For the entire sample we then used this probability to draw whether or not the particular sample was non-zero, and if so we sampled without replacement a non-zero counts from the existing data. At some point the number of non-zero counts available for sampling are depleted (as we increased the probability of non-zero samples) and the remaining samples are assigned zero’s. This implies that the counts remain the same but get shuffled so that the non-zero counts are more likely to occur in a sample where this taxon is differentially abundant. Each sample in this group then has an increased probability of a non-zero count, that is further multiplied by the effect size used.

#### Adding covariates

We made a similar simulated data set containing confounding factors. In addition to the diet, we included two additional simulated covariates for every subject: Urbanization (low/high) and Age (20–69). The effect of urbanization was simulated like that of diet: subjects were allocated to low/high urbanization and 10% of the taxa were made differentially abundant in both groups with an effect size +200%. Ages of 20, 21,..., 69 were allocated to each subject and a differential effect was added for 10% of taxa with the effect depending linearly on age from 0% to 400%. These effects increase both the counts and the odds of non-zero counts. So there are three sources of signal to disentangle: different 10% of taxa are differentially abundant for each diet group, urbanization, and affected by age.

In order to act as confounders, urbanization and age need to be correlated to the diet group. Table 3 shows the probability of being assigned to a joint Diet and Urbanization group used to produce such a correlation, and Table 4 shows the probability of being assigned into a particular age range given diet group. We uniformly assigned age within this age range. Some taxa might now be detected as differentially abundant, not because the diet really influenced them, but because they also tended to have a different degree of urbanization and age.

**Table 3:** Joint probability of diet and urbanization

Urbanization	Diet			
	Meateater (%)	Fisheater (%)	Vegetarian (%)	Vegan (%)
Low	20	15	10	5
High	5	10	15	20

**Table 4:** Conditional probability of age given diet

Age	Diet			
	Meateater (%)	Fisheater (%)	Vegetarian (%)	Vegan (%)
[20,30)	0	10	30	40
[30,40)	10	15	25	30
[40,50)	20	20	20	20
[50,60)	30	25	15	10
[60,70)	40	30	10	0

All experiments were run in parallel on a high-performance RedHat 7.9 LSF Linux cluster with R version 4.0.5. Each experiment was run 50 times.

## Results

We first compare the 4 diet groups (meateater, fisheater, vegetarian, vegan) in the simulations without confounding factors, and then add Urbanization (low/high) and Age (20–60) as confounding factors. To both data sets we either introduce signal only in the counts, or in both counts & zeros.

In each experiment, we compare the likelihood model and the PRR-test by presenting the following four metrics:

1. True Positive Rate (TPR) at a *p* value = 0.05 threshold.
2. False Positive Rate (FPR) at a *p* value = 0.05 threshold.
3. Power when the *p* value is chosen such that true FPR = 0.05 (power@0.05),
4. Area Under the ROC Curve up to the FPR = 0.10 (AUC@0.10), normalized by the maximum area attainable.

**Fig. 2:**
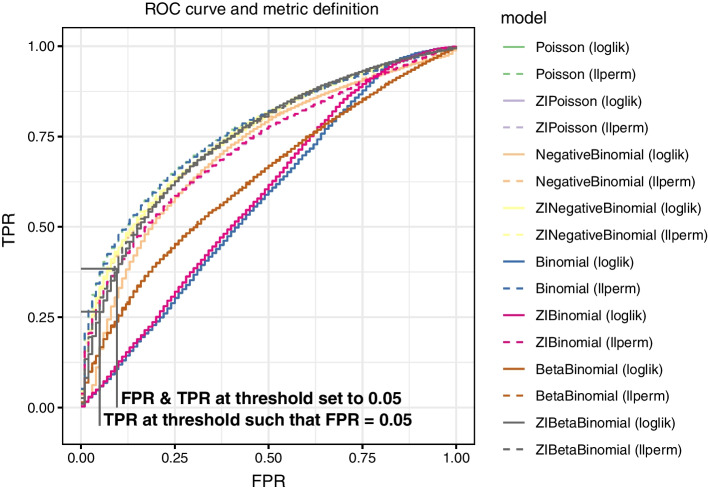
ROC curve on diet groups with confounding variables, signal in both counts & zeros. ZIBetaBinomial (loglik) has a TPR 0.38 and 0.10 FPR at a 0.05 *p* value threshold. If we set the threshold such that FPR equals 0.05, the TPR@0.05 is 0.27. We can similarly calculate the AUC@0.10 as area under TPR over FPR values 0.00–0.10

These are illustrated by the ROC curve in Fig. 2. Note that power@0.05 and AUC@0.10 can not be calculated in real data, because we cannot set the threshold at a given FPR rate without knowing the truly differentially abundant taxa, but can be calculated from simulations.

### Group comparison without confounding

For the first experiment, we aim to detect taxa that are differentially abundant in a comparison of Diet groups in a situation without confounding variables. The likelihood and PRR-test based model statistics are shown in Table 5 for signal in counts and Table 6 for signal in counts & zeros, both using an effect size +100%.

**Table 5:** Model comparison on diet groups (signal in counts, effect size + 100%)

Family	Type	Power	FPR	Power@0.05	AUC@0.10
Poisson	(loglik)	0.99 ($\pm 0.00$)	0.98 ($\pm 0.00$)	0.09 ($\pm 0.00$)	0.52 ($\pm 0.01$)
Poisson	(llperm)	0.29 ($\pm 0.01$)	0.05 ($\pm 0.00$)	0.28 ($\pm 0.01$)	0.72 ($\pm 0.01$)
ZIPoisson	(loglik)	0.99 ($\pm 0.00$)	0.93 ($\pm 0.00$)	0.11 ($\pm 0.00$)	0.52 ($\pm 0.01$)
ZIPoisson	(llperm)	0.40 ($\pm 0.00$)	0.05 ($\pm 0.00$)	0.40 ($\pm 0.01$)	0.77 ($\pm 0.01$)
NegativeBinomial	(loglik)	0.11 ($\pm 0.00$)	0.02 ($\pm 0.00$)	0.21 ($\pm 0.01$)	0.66 ($\pm 0.01$)
NegativeBinomial	(llperm)	0.26 ($\pm 0.00$)	0.05 ($\pm 0.00$)	0.26 ($\pm 0.01$)	0.70 ($\pm 0.01$)
ZINegativeBinomial	(loglik)	0.49 ($\pm 0.01$)	0.15 ($\pm 0.00$)	0.28 ($\pm 0.01$)	0.63 ($\pm 0.01$)
ZINegativeBinomial	(llperm)	0.34 ($\pm 0.00$)	0.05 ($\pm 0.00$)	0.34 ($\pm 0.01$)	0.75 ($\pm 0.01$)
Binomial	(loglik)	0.99 ($\pm 0.00$)	0.98 ($\pm 0.00$)	0.09 ($\pm 0.00$)	0.52 ($\pm 0.01$)
Binomial	(llperm)	0.29 ($\pm 0.01$)	0.05 ($\pm 0.00$)	0.28 ($\pm 0.01$)	0.72 ($\pm 0.01$)
ZIBinomial	(loglik)	0.99 ($\pm 0.00$)	0.93 ($\pm 0.00$)	0.11 ($\pm 0.00$)	0.52 ($\pm 0.01$)
ZIBinomial	(llperm)	0.41 ($\pm 0.00$)	0.05 ($\pm 0.00$)	0.40 ($\pm 0.01$)	0.76 ($\pm 0.01$)
BetaBinomial	(loglik)	0.14 ($\pm 0.00$)	0.09 ($\pm 0.00$)	0.09 ($\pm 0.00$)	0.59 ($\pm 0.01$)
BetaBinomial	(llperm)	0.08 ($\pm 0.00$)	0.05 ($\pm 0.00$)	0.08 ($\pm 0.00$)	0.59 ($\pm 0.01$)
ZIBetaBinomial	(loglik)	0.33 ($\pm 0.01$)	0.05 ($\pm 0.00$)	0.33 ($\pm 0.01$)	0.71 ($\pm 0.01$)
ZIBetaBinomial	(llperm)	0.34 ($\pm 0.01$)	0.05 ($\pm 0.00$)	0.35 ($\pm 0.01$)	0.74 ($\pm 0.01$)

**Table 6:** Model comparison on diet groups (signal in counts & zeros, effect size +100%)

Family	Type	Power	FPR	Power@0.05	AUC@0.10
Poisson	(loglik)	1.00 ($\pm 0.00$)	0.98 ($\pm 0.00$)	0.12 ($\pm 0.00$)	0.54 ($\pm 0.01$)
Poisson	(llperm)	0.49 ($\pm 0.01$)	0.05 ($\pm 0.00$)	0.48 ($\pm 0.01$)	0.78 ($\pm 0.01$)
ZIPoisson	(loglik)	0.99 ($\pm 0.00$)	0.93 ($\pm 0.00$)	0.11 ($\pm 0.00$)	0.53 ($\pm 0.01$)
ZIPoisson	(llperm)	0.39 ($\pm 0.01$)	0.05 ($\pm 0.00$)	0.39 ($\pm 0.01$)	0.75 ($\pm 0.01$)
NegativeBinomial	(loglik)	0.17 ($\pm 0.00$)	0.02 ($\pm 0.00$)	0.32 ($\pm 0.01$)	0.66 ($\pm 0.01$)
NegativeBinomial	(llperm)	0.44 ($\pm 0.01$)	0.05 ($\pm 0.00$)	0.43 ($\pm 0.01$)	0.73 ($\pm 0.01$)
ZINegativeBinomial	(loglik)	0.59 ($\pm 0.00$)	0.15 ($\pm 0.00$)	0.37 ($\pm 0.01$)	0.69 ($\pm 0.01$)
ZINegativeBinomial	(llperm)	0.42 ($\pm 0.00$)	0.05 ($\pm 0.00$)	0.42 ($\pm 0.01$)	0.74 ($\pm 0.01$)
Binomial	(loglik)	1.00 ($\pm 0.00$)	0.98 ($\pm 0.00$)	0.12 ($\pm 0.00$)	0.54 ($\pm 0.01$)
Binomial	(llperm)	0.49 ($\pm 0.01$)	0.05 ($\pm 0.00$)	0.48 ($\pm 0.01$)	0.78 ($\pm 0.01$)
ZIBinomial	(loglik)	0.99 ($\pm 0.00$)	0.93 ($\pm 0.00$)	0.11 ($\pm 0.00$)	0.53 ($\pm 0.01$)
ZIBinomial	(llperm)	0.39 ($\pm 0.00$)	0.05 ($\pm 0.00$)	0.39 ($\pm 0.01$)	0.75 ($\pm 0.01$)
BetaBinomial	(loglik)	0.28 ($\pm 0.01$)	0.10 ($\pm 0.00$)	0.19 ($\pm 0.01$)	0.64 ($\pm 0.01$)
BetaBinomial	(llperm)	0.20 ($\pm 0.01$)	0.05 ($\pm 0.00$)	0.20 ($\pm 0.01$)	0.64 ($\pm 0.01$)
ZIBetaBinomial	(loglik)	0.39 ($\pm 0.00$)	0.05 ($\pm 0.00$)	0.39 ($\pm 0.01$)	0.72 ($\pm 0.01$)
ZIBetaBinomial	(llperm)	0.41 ($\pm 0.00$)	0.05 ($\pm 0.00$)	0.41 ($\pm 0.01$)	0.73 ($\pm 0.01$)

Most likelihood based models without overdispersion have high false positive rates: over 90% of non-differentially abundant taxa are detected as false positives for (ZI)Binomial and (ZI) Poisson distributions. Overdispersed models do better, but still have too high false positive rates. Only the ZIBetaBinomial model produced the correct nominal 5% FPR, while having the power to detect 33% (counts) or 39% (counts &zeros) of the differentially abundant taxa. The PRR-test based models all had the correct nominal 5% FPR rate, and the zero-inflated models all had power of 34–41% (counts) or 39%-42% (counts &zeros) to detect the taxa. In a more realistic setting where signal occurs in both counts and zeros, the standard Binomial and Poisson models based on likelihood perform very poorly but become effective with the PRR-test, achieving 49% power and 5% FPR.

**Fig. 4:**
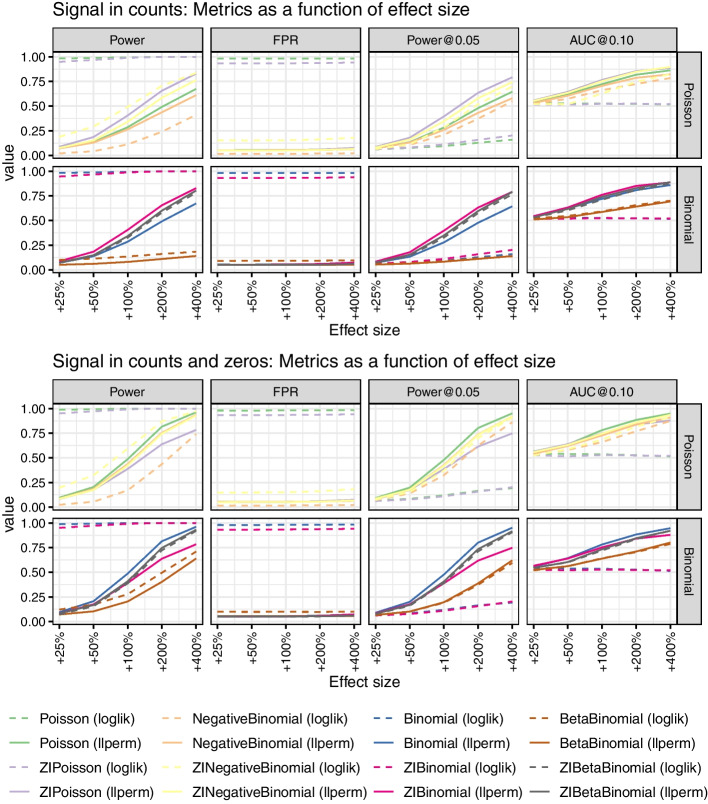
Model comparison on diet groups for different effect sizes (+ 25%, + 50%, + 100%, + 200%, + 400%)

Figure 4 in the “Appendix” shows that these findings are consistent with different effect sizes: a models’ power increases as the effect size increases, but the PRR-test based models maintain the correct nominal FPR, while likelihood based models maintain the high rate of false positives.

### Group comparison with confounding

For the second experiment, we aim to detect taxa that are differentially abundant between Diet groups in a situation with confounding variables. Table 7 shows the results in the experiments with signal in counts and Table 8 those for signal in counts & zeros both using effect size +100%.

**Table 7:** Model comparison on diet groups with confounding variables (signal in counts, effect size +100%)

Family	Type	Power	FPR	Power@0.05	AUC@0.10
Poisson	(loglik)	0.99 ($\pm 0.00$)	0.98 ($\pm 0.00$)	0.09 ($\pm 0.00$)	0.53 ($\pm 0.01$)
Poisson	(llperm)	0.22 ($\pm 0.01$)	0.05 ($\pm 0.00$)	0.22 ($\pm 0.01$)	0.69 ($\pm 0.01$)
ZIPoisson	(loglik)	0.99 ($\pm 0.00$)	0.94 ($\pm 0.00$)	0.10 ($\pm 0.01$)	0.53 ($\pm 0.01$)
ZIPoisson	(llperm)	0.32 ($\pm 0.01$)	0.05 ($\pm 0.00$)	0.32 ($\pm 0.01$)	0.72 ($\pm 0.01$)
NegativeBinomial	(loglik)	0.11 ($\pm 0.00$)	0.04 ($\pm 0.00$)	0.15 ($\pm 0.01$)	0.56 ($\pm 0.01$)
NegativeBinomial	(llperm)	0.22 ($\pm 0.01$)	0.06 ($\pm 0.00$)	0.21 ($\pm 0.01$)	0.66 ($\pm 0.01$)
ZINegativeBinomial	(loglik)	0.46 ($\pm 0.01$)	0.16 ($\pm 0.00$)	0.27 ($\pm 0.01$)	0.69 ($\pm 0.01$)
ZINegativeBinomial	(llperm)	0.29 ($\pm 0.01$)	0.05 ($\pm 0.00$)	0.29 ($\pm 0.01$)	0.72 ($\pm 0.01$)
Binomial	(loglik)	0.99 ($\pm 0.00$)	0.98 ($\pm 0.00$)	0.09 ($\pm 0.00$)	0.53 ($\pm 0.01$)
Binomial	(llperm)	0.22 ($\pm 0.01$)	0.05 ($\pm 0.00$)	0.22 ($\pm 0.01$)	0.69 ($\pm 0.01$)
ZIBinomial	(loglik)	0.99 ($\pm 0.00$)	0.94 ($\pm 0.00$)	0.10 ($\pm 0.01$)	0.53 ($\pm 0.01$)
ZIBinomial	(llperm)	0.32 ($\pm 0.01$)	0.05 ($\pm 0.00$)	0.32 ($\pm 0.01$)	0.73 ($\pm 0.01$)
BetaBinomial	(loglik)	0.14 ($\pm 0.01$)	0.11 ($\pm 0.00$)	0.08 ($\pm 0.00$)	0.59 ($\pm 0.01$)
BetaBinomial	(llperm)	0.08 ($\pm 0.00$)	0.06 ($\pm 0.00$)	0.07 ($\pm 0.00$)	0.58 ($\pm 0.01$)
ZIBetaBinomial	(loglik)	0.34 ($\pm 0.01$)	0.10 ($\pm 0.00$)	0.23 ($\pm 0.01$)	0.64 ($\pm 0.01$)
ZIBetaBinomial	(llperm)	0.26 ($\pm 0.00$)	0.05 ($\pm 0.00$)	0.26 ($\pm 0.01$)	0.68 ($\pm 0.01$)

**Table 8:** Model comparison on diet groups with confounding variables (signal in counts & zeros, effect size +100%)

Family	Type	Power	FPR	Power@0.05	AUC@0.10
Poisson	(loglik)	1.00 ($\pm 0.00$)	0.99 ($\pm 0.00$)	0.11 ($\pm 0.00$)	0.53 ($\pm 0.01$)
Poisson	(llperm)	0.37 ($\pm 0.01$)	0.05 ($\pm 0.00$)	0.37 ($\pm 0.01$)	0.74 ($\pm 0.01$)
ZIPoisson	(loglik)	0.99 ($\pm 0.00$)	0.94 ($\pm 0.00$)	0.10 ($\pm 0.01$)	0.54 ($\pm 0.01$)
ZIPoisson	(llperm)	0.30 ($\pm 0.01$)	0.05 ($\pm 0.00$)	0.30 ($\pm 0.01$)	0.72 ($\pm 0.01$)
NegativeBinomial	(loglik)	0.17 ($\pm 0.00$)	0.05 ($\pm 0.00$)	0.16 ($\pm 0.01$)	0.48 ($\pm 0.01$)
NegativeBinomial	(llperm)	0.34 ($\pm 0.01$)	0.05 ($\pm 0.00$)	0.33 ($\pm 0.01$)	0.71 ($\pm 0.01$)
ZINegativeBinomial	(loglik)	0.52 ($\pm 0.01$)	0.15 ($\pm 0.00$)	0.33 ($\pm 0.01$)	0.70 ($\pm 0.01$)
ZINegativeBinomial	(llperm)	0.34 ($\pm 0.01$)	0.05 ($\pm 0.00$)	0.34 ($\pm 0.01$)	0.71 ($\pm 0.01$)
Binomial	(loglik)	1.00 ($\pm 0.00$)	0.99 ($\pm 0.00$)	0.11 ($\pm 0.00$)	0.53 ($\pm 0.01$)
Binomial	(llperm)	0.37 ($\pm 0.01$)	0.05 ($\pm 0.00$)	0.38 ($\pm 0.01$)	0.74 ($\pm 0.01$)
ZIBinomial	(loglik)	0.99 ($\pm 0.00$)	0.94 ($\pm 0.00$)	0.10 ($\pm 0.01$)	0.54 ($\pm 0.01$)
ZIBinomial	(llperm)	0.30 ($\pm 0.01$)	0.05 ($\pm 0.00$)	0.30 ($\pm 0.01$)	0.72 ($\pm 0.01$)
BetaBinomial	(loglik)	0.26 ($\pm 0.00$)	0.10 ($\pm 0.00$)	0.17 ($\pm 0.00$)	0.63 ($\pm 0.01$)
BetaBinomial	(llperm)	0.17 ($\pm 0.00$)	0.05 ($\pm 0.00$)	0.17 ($\pm 0.00$)	0.63 ($\pm 0.01$)
ZIBetaBinomial	(loglik)	0.38 ($\pm 0.01$)	0.10 ($\pm 0.00$)	0.27 ($\pm 0.01$)	0.63 ($\pm 0.01$)
ZIBetaBinomial	(llperm)	0.30 ($\pm 0.01$)	0.05 ($\pm 0.00$)	0.30 ($\pm 0.01$)	0.67 ($\pm 0.01$)

**Fig. 5:**
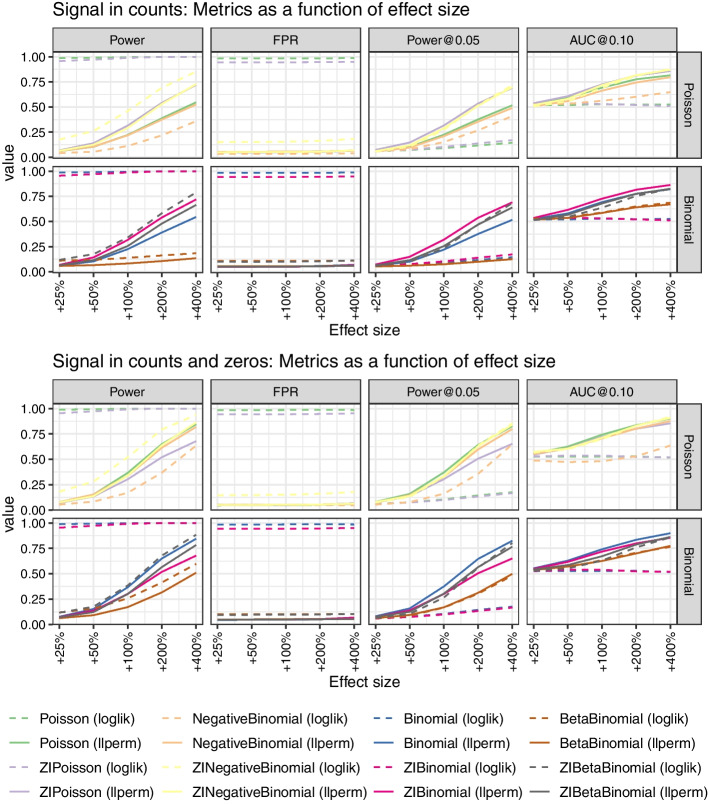
Model comparison on diet groups with confounding variables for different effect sizes (+ 25%, + 50%, + 100%, + 200%, + 400%)

As expected, the models lose some power when additional covariates are introduced. Of the likelihood based models, only the Negative Binomial had a correct nominal 5% FPR rate with a power of 11% (counts) or 17% (counts &zeros). The PRR-test based models all had the correct nominal 5% FPR rate, and the zero-inflated models had power of 26–32% (counts) or 30–34% (counts &zeros). When there is signal in both counts and zeros, again the standard Binomial and Poisson models based on likelihood perform very poorly but become effective with the PRR-test, achieving 37% power and 5% FPR. In Fig. 5 in the “Appendix” shows again the the results are consistent with different effect sizes.

## Discussion

Our simulations show that the PRR-test—as expected—controls the FPR, but also seems to improve the power in a regression setting. Models with overdispersion and zero-inflation are generally better in the likelihood setting, but differences are less pronounced in PRR-test based approaches. Surprisingly, in a more realistic setting where the signal in counts co-occurs with signal in zeros—both in the same direction -, the PRR-test makes even the standard Poisson and Binomial models perform well. It seems that zero-inflated models are most needed if a signal has been introduced only to counts, because the random variation in the occurrence of non-zero counts tends to obfuscate the signal, making the models without zero-inflation lose power.

The results generally align with previous literature, except for two findings. First, the standard Negative Binomial seems to have too low FPR (0.02) in the comparison of groups without confounding. We investigated that this seemed to be caused by some taxas having very high zero-inflation in our data. With a better fitting zero-inflated Negative Binomial model we did observe the expected too high FPR. Also when we simulated data from Negative Binomial (instead of simulations based on real data) we observed a too high FPR. Second, the standard Beta Binomial has a very low power and results differ - due to different assumptions on overdispersion—from that of using the Negative Binomial. We found that this model also has significant problems with excess zeros which regularly cause numerical problems. Sometimes the likelihood cannot even be evaluated outside a narrow neighbourhood of the solution, necessitating very accurate starting values for the optimization process.

One surprise in doing this work was that the glm.nb function from the MASS package converged to a different solution compared to our likelihood based implementation in some of the datasets (Comparing packages in “Appendix”). The divergent glm.nb solutions had either very small or very large *p* value, and was caused by a lack of convergence of the estimate of the overdispersion parameter, which went unnoticed as the function returned a converged status. This made the FPR even larger than with our implementation. With the exception of this issue with MASS, our results tended to be identical to those delivered by other packages.

We argued that simulating data by resampling a real data set provides more realistic results than simulating data from a known statistical distribution. However, our simulations are based on a single dataset. This might not fully reflect all possible data in microbiome studies. Also we assumed the original data set did not contain signal, so the data used for simulation might be more overdispersed than data that are truly without signal. Also, adding signal by multiplying the counts will increase the variance in simulated data. Nevertheless we believe our simulation gives a good indication of the relative merits of the different methods. We publish the data set, a reproducible R Markdown source code for the simulation experiments, and a simple implementation of the method in the “Appendix”.

## Conclusion

The PRR-test was shown to provide useful new tools for microbiome data analysis. Standard regression models based on it are able to maintain the correct nominal false positive rate expected from the null hypothesis, while having equal or greater power to detect the true positives as models based on likelihood at a given false positive rate. Likelihood models can have a high rate of false positives and it is not possible to adjust for this in real data sets where the ground truth is unknown. This method therefore provides a new approach which is competitive in power, but also offer insurance against model misspecification. As standard models may not provide a good fit to data, so such robustness can be viewed as a major benefit.

## Figures and Tables

**Fig. 3:**
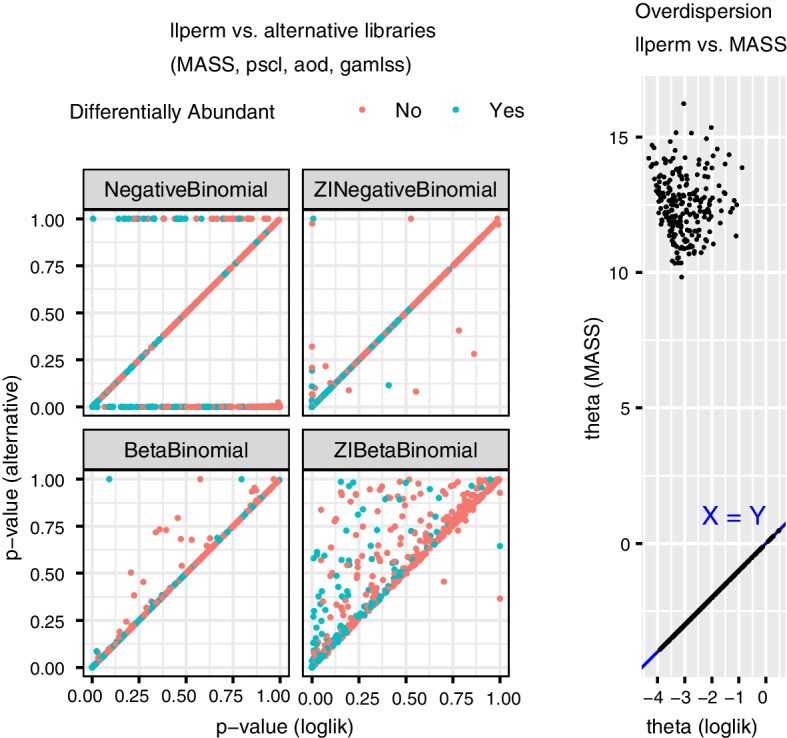
*p* values from our implementation compared to those of other packages for taxa with and without signal (left); theta estimates from our implementation compared to those of glm.nb from MASS (right). Points in the cloud at the top indicating non-convergence of theta are the points delivering different *p* values

## Data Availability

The dataset and the source code for the experiments of this article are available in the repository ‘llperm’: https://github.com/majuvi/llperm

## Appendix

### Using our package

We extend the original glmperm function (prr.test) and implemented one new function for GLMs (prr.test.glm) and another for likelihood models (prr.test.ll). Example:

### Comparing packages

We verified our implementation by comparing it with other R-packages. Our results were virtual identical to likelihood based methods, and other methods tended to give the same results for almost all taxa. Exception was glm.nb from the MASS package, which produced many *p* values close to 0 or 1 but otherwise agreed with our method. This is illustrated in Fig. 3.

Upon investigating the reason, we found that MASS estimates the Negative Binomial distribution parameters in a two-stage process whereby first the parameters are estimated for a fixed overdispersion parameter and then the overdispersion is estimated given these parameters. This process did not converge for all taxa and sometimes indicated significant underdispersion, where overdispersion is 1/exp(theta), as illustrated by the very high thetas in Fig. 3. Increasing the number of iterations would eventually crash the estimation procedure. Otherwise the package gave identical *p* values and estimates of theta as indicated by the blue reference line. Our implementation tends to have equal or better power/AUC than functions from other packages, as seen from the similar or lower *p* values for taxa with signal (Fig. 3(left)).

An example of data for a taxon that causes this problem is given in Table 9 and the associated simple code listing below. Although the problem tends to occur in taxa with many zero-counts, it can also occur with non-zero counts if most counts are low but some are very high.

**Table 9:** Example: table of counts where MASS diverges

Count	0	6	10	11	12	13	16	20	21	23	26	27	28	32	33	39	65
n	125	2	3	3	1	2	1	2	1	2	1	1	1	1	1	1	1

### Comparing alternative approaches

We briefly compared our proposed models to alternative approaches and across data sets with different properties. Due to limited sample sizes and support in other packages, for these experiments we combined the four diet groups (meateater, fisheater, vegan, vegetarian) into two (vegY, vegN) and used a similar confounding structure as in Tables 3 and 4. The counts in VEGA dataset were then modified with an otherwise identical simulation.

We compared the permutation based Poisson family count regression models (Poisson, ZIPoisson, NegativeBinomial, ZINegativeBinomial) to alternative approaches. There are several other widely applicable models that have also indicated correct false positive rate control: ALDEx2, ANCOM-BC, LinDA, and Maaslin2, for example [26, 27]. For an alternative to the regression approach, we also performed a stratified Fisher-Pitman and Kurskal–Wallis permutation tests using the ‘coin’ R package, where every combination of the covariates defines a separate strata [17]. The results for different models in resampled VEGA data are displayed in Table 10. The alternative approaches indicate lower than expected false positive rates and have a considerably lower power, as we discussed in connection to the Negative Binomial distribution. DESeq2 and EdgeR deliver very similar results to Negative Binomial count regression in our implementation, which is to be expected because they only differ from our implementation in the estimation of the taxon dispersion parameters. In the VEGA data set ‘llperm’ compares very favorably to alternative approaches, which are outperformed by a simple stratified permutation test in ‘coin’.

**Table 10:** Model comparison and alternative approaches (signal in counts, effect size +100%))

Family	Type	Power	FPR	Power@0.05	AUC@0.10
DESeq2	(baseline)	0.11 ($\pm 0.00$)	0.02 ($\pm 0.00$)	0.17 ($\pm 0.01$)	0.62 ($\pm 0.01$)
EdgeR	(baseline)	0.09 ($\pm 0.00$)	0.01 ($\pm 0.00$)	0.19 ($\pm 0.01$)	0.66 ($\pm 0.01$)
ALDEx2	(baseline)	0.04 ($\pm 0.00$)	0.01 ($\pm 0.00$)	0.09 ($\pm 0.00$)	0.59 ($\pm 0.01$)
ANCOMBC	(baseline)	0.11 ($\pm 0.00$)	0.06 ($\pm 0.00$)	0.10 ($\pm 0.00$)	0.59 ($\pm 0.01$)
LinDA	(baseline)	0.10 ($\pm 0.00$)	0.05 ($\pm 0.00$)	0.10 ($\pm 0.00$)	0.59 ($\pm 0.01$)
Maaslin2 (lm)	(baseline)	0.10 ($\pm 0.00$)	0.05 ($\pm 0.00$)	0.10 ($\pm 0.00$)	0.60 ($\pm 0.01$)
kruskal_test (strata)	(baseline)	0.12 ($\pm 0.01$)	0.05 ($\pm 0.00$)	0.12 ($\pm 0.00$)	0.62 ($\pm 0.01$)
oneway_test (strata)	(baseline)	0.18 ($\pm 0.01$)	0.05 ($\pm 0.00$)	0.18 ($\pm 0.01$)	0.65 ($\pm 0.01$)
Poisson	(llperm)	0.22 ($\pm 0.01$)	0.05 ($\pm 0.00$)	0.21 ($\pm 0.01$)	0.66 ($\pm 0.01$)
Poisson	(loglik)	0.92 ($\pm 0.00$)	0.85 ($\pm 0.00$)	0.14 ($\pm 0.01$)	0.54 ($\pm 0.01$)
ZIPoisson	(llperm)	0.29 ($\pm 0.01$)	0.05 ($\pm 0.00$)	0.29 ($\pm 0.01$)	0.70 ($\pm 0.01$)
ZIPoisson	(loglik)	0.92 ($\pm 0.00$)	0.78 ($\pm 0.00$)	0.16 ($\pm 0.01$)	0.57 ($\pm 0.01$)
NegativeBinomial	(llperm)	0.23 ($\pm 0.01$)	0.06 ($\pm 0.00$)	0.22 ($\pm 0.01$)	0.69 ($\pm 0.01$)
NegativeBinomial	(loglik)	0.13 ($\pm 0.00$)	0.03 ($\pm 0.00$)	0.18 ($\pm 0.01$)	0.60 ($\pm 0.01$)
ZINegativeBinomial	(llperm)	0.29 ($\pm 0.01$)	0.05 ($\pm 0.00$)	0.29 ($\pm 0.01$)	0.70 ($\pm 0.01$)
ZINegativeBinomial	(loglik)	0.39 ($\pm 0.01$)	0.10 ($\pm 0.00$)	0.27 ($\pm 0.01$)	0.68 ($\pm 0.01$)

### Comparing different data sets

We briefly compared different data sets to assess how universal the results are and whether they are impacted by the simulation method. Three data sets come from the ‘mia’ package: soilrep (56 × 16,825), enterotype (280 × 553), dmn_se (278 × 130), where the (sample size × number of OTUs) is given in parenthesis. We filtered these into OTUs with at least 5% prevalence. Simulated signal was added using the same resampling scheme as with VEGA data. The ‘dmn_se’ dataset is based on a Dirichlet Multinomial distribution. We obtained another three data sets by simulating data directly from parametric distributions: sparseDOSSA [28] (180 × 200), metaSPARSim [29] (80 × 758), VegaZINB (149 × 531). The ‘sparseDOSSA’ package uses a truncated zero-inflated log-normal distribution. The ‘sparseDOSSA’ package uses a multivariate hypergeometric distribution with parameters based on the Human Microbiome Project data set. The third data set was generated by fitting zero-inflated negative binomial distributions for each taxa to our VEGA data, then generating a data set with same dimensions from the fitted distributions. Signal was added to the data set by modifying the parameters of these distributions.

**Table 11:** Model comparison on different data sets (signal in counts, effect size +100%)

Data Set	Family	Type	Power	FPR	Power@0.05	AUC@0.10
soilrep	Poisson	(loglik)	0.53 ($\pm 0.00$)	0.21 ($\pm 0.01$)	0.29 ($\pm 0.01$)	0.69 ($\pm 0.01$)
soilrep	Poisson	(llperm)	0.17 ($\pm 0.01$)	0.05 ($\pm 0.00$)	0.18 ($\pm 0.01$)	0.62 ($\pm 0.01$)
soilrep	ZIPoisson	(loglik)	0.54 ($\pm 0.01$)	0.17 ($\pm 0.01$)	0.34 ($\pm 0.01$)	0.71 ($\pm 0.01$)
soilrep	ZIPoisson	(llperm)	0.30 ($\pm 0.01$)	0.05 ($\pm 0.00$)	0.30 ($\pm 0.01$)	0.69 ($\pm 0.01$)
soilrep	NegativeBinomial	(loglik)	0.18 ($\pm 0.01$)	0.06 ($\pm 0.00$)	0.15 ($\pm 0.01$)	0.59 ($\pm 0.02$)
soilrep	NegativeBinomial	(llperm)	0.17 ($\pm 0.01$)	0.05 ($\pm 0.00$)	0.17 ($\pm 0.01$)	0.61 ($\pm 0.01$)
soilrep	ZINegativeBinomial	(loglik)	0.30 ($\pm 0.01$)	0.05 ($\pm 0.00$)	0.29 ($\pm 0.00$)	0.70 ($\pm 0.01$)
soilrep	ZINegativeBinomial	(llperm)	0.27 ($\pm 0.01$)	0.05 ($\pm 0.00$)	0.28 ($\pm 0.01$)	0.67 ($\pm 0.01$)
enterotype	Poisson	(loglik)	1.00 ($\pm 0.00$)	0.97 ($\pm 0.01$)	0.11 ($\pm 0.01$)	0.56 ($\pm 0.01$)
enterotype	Poisson	(llperm)	0.62 ($\pm 0.02$)	0.09 ($\pm 0.02$)	0.70 ($\pm 0.02$)	0.89 ($\pm 0.01$)
enterotype	ZIPoisson	(loglik)	1.00 ($\pm 0.00$)	0.95 ($\pm 0.01$)	0.12 ($\pm 0.01$)	0.56 ($\pm 0.02$)
enterotype	ZIPoisson	(llperm)	0.81 ($\pm 0.01$)	0.10 ($\pm 0.02$)	0.78 ($\pm 0.02$)	0.92 ($\pm 0.01$)
enterotype	NegativeBinomial	(loglik)	0.09 ($\pm 0.01$)	0.02 ($\pm 0.00$)	0.40 ($\pm 0.02$)	0.53 ($\pm 0.02$)
enterotype	NegativeBinomial	(llperm)	0.67 ($\pm 0.02$)	0.07 ($\pm 0.02$)	0.75 ($\pm 0.02$)	0.90 ($\pm 0.01$)
enterotype	ZINegativeBinomial	(loglik)	0.84 ($\pm 0.01$)	0.14 ($\pm 0.02$)	0.76 ($\pm 0.02$)	0.81 ($\pm 0.01$)
enterotype	ZINegativeBinomial	(llperm)	0.81 ($\pm 0.01$)	0.07 ($\pm 0.02$)	0.81 ($\pm 0.01$)	0.93 ($\pm 0.01$)
dmn_se	Poisson	(loglik)	0.94 ($\pm 0.01$)	0.72 ($\pm 0.01$)	0.40 ($\pm 0.02$)	0.83 ($\pm 0.02$)
dmn_se	Poisson	(llperm)	0.51 ($\pm 0.02$)	0.08 ($\pm 0.01$)	0.48 ($\pm 0.02$)	0.87 ($\pm 0.02$)
dmn_se	ZIPoisson	(loglik)	0.93 ($\pm 0.01$)	0.68 ($\pm 0.01$)	0.40 ($\pm 0.02$)	0.81 ($\pm 0.02$)
dmn_se	ZIPoisson	(llperm)	0.58 ($\pm 0.02$)	0.07 ($\pm 0.01$)	0.55 ($\pm 0.02$)	0.88 ($\pm 0.02$)
dmn_se	NegativeBinomial	(loglik)	0.56 ($\pm 0.02$)	0.13 ($\pm 0.01$)	0.42 ($\pm 0.03$)	0.78 ($\pm 0.02$)
dmn_se	NegativeBinomial	(llperm)	0.56 ($\pm 0.02$)	0.08 ($\pm 0.01$)	0.53 ($\pm 0.02$)	0.87 ($\pm 0.02$)
dmn_se	ZINegativeBinomial	(loglik)	0.59 ($\pm 0.02$)	0.11 ($\pm 0.01$)	0.48 ($\pm 0.02$)	0.83 ($\pm 0.02$)
dmn_se	ZINegativeBinomial	(llperm)	0.58 ($\pm 0.02$)	0.07 ($\pm 0.01$)	0.55 ($\pm 0.02$)	0.89 ($\pm 0.02$)
sparseDOSSA	Poisson	(loglik)	0.98 ($\pm 0.00$)	0.87 ($\pm 0.00$)	0.50 ($\pm 0.01$)	0.78 ($\pm 0.01$)
sparseDOSSA	Poisson	(llperm)	0.58 ($\pm 0.02$)	0.07 ($\pm 0.00$)	0.51 ($\pm 0.02$)	0.73 ($\pm 0.01$)
sparseDOSSA	ZIPoisson	(loglik)	1.00 ($\pm 0.00$)	0.73 ($\pm 0.01$)	0.83 ($\pm 0.01$)	0.85 ($\pm 0.01$)
sparseDOSSA	ZIPoisson	(llperm)	1.00 ($\pm 0.00$)	0.09 ($\pm 0.00$)	0.99 ($\pm 0.00$)	0.93 ($\pm 0.01$)
sparseDOSSA	NegativeBinomial	(loglik)	0.04 ($\pm 0.01$)	0.01 ($\pm 0.00$)	0.33 ($\pm 0.02$)	0.61 ($\pm 0.02$)
sparseDOSSA	NegativeBinomial	(llperm)	0.58 ($\pm 0.02$)	0.06 ($\pm 0.00$)	0.52 ($\pm 0.02$)	0.74 ($\pm 0.01$)
sparseDOSSA	ZINegativeBinomial	(loglik)	1.00 ($\pm 0.00$)	0.12 ($\pm 0.00$)	0.96 ($\pm 0.01$)	0.91 ($\pm 0.01$)
sparseDOSSA	ZINegativeBinomial	(llperm)	1.00 ($\pm 0.00$)	0.09 ($\pm 0.00$)	0.98 ($\pm 0.01$)	0.89 ($\pm 0.01$)
metaSPARSim	Poisson	(loglik)	0.72 ($\pm 0.01$)	0.48 ($\pm 0.00$)	0.11 ($\pm 0.01$)	0.61 ($\pm 0.02$)
metaSPARSim	Poisson	(llperm)	0.36 ($\pm 0.01$)	0.06 ($\pm 0.00$)	0.33 ($\pm 0.02$)	0.72 ($\pm 0.02$)
metaSPARSim	ZIPoisson	(loglik)	0.64 ($\pm 0.01$)	0.42 ($\pm 0.00$)	0.10 ($\pm 0.01$)	0.62 ($\pm 0.02$)
metaSPARSim	ZIPoisson	(llperm)	0.32 ($\pm 0.02$)	0.06 ($\pm 0.00$)	0.31 ($\pm 0.02$)	0.75 ($\pm 0.02$)
metaSPARSim	NegativeBinomial	(loglik)	0.38 ($\pm 0.01$)	0.07 ($\pm 0.00$)	0.34 ($\pm 0.02$)	0.73 ($\pm 0.01$)
metaSPARSim	NegativeBinomial	(llperm)	0.36 ($\pm 0.01$)	0.06 ($\pm 0.00$)	0.34 ($\pm 0.02$)	0.73 ($\pm 0.01$)
metaSPARSim	ZINegativeBinomial	(loglik)	0.31 ($\pm 0.01$)	0.06 ($\pm 0.00$)	0.30 ($\pm 0.02$)	0.70 ($\pm 0.02$)
metaSPARSim	ZINegativeBinomial	(llperm)	0.34 ($\pm 0.01$)	0.06 ($\pm 0.00$)	0.32 ($\pm 0.02$)	0.73 ($\pm 0.02$)
VegaZINB	Poisson	(loglik)	0.99 ($\pm 0.00$)	0.82 ($\pm 0.00$)	0.44 ($\pm 0.01$)	0.64 ($\pm 0.01$)
VegaZINB	Poisson	(llperm)	0.80 ($\pm 0.01$)	0.05 ($\pm 0.00$)	0.80 ($\pm 0.01$)	0.89 ($\pm 0.00$)
VegaZINB	ZIPoisson	(loglik)	0.98 ($\pm 0.00$)	0.72 ($\pm 0.00$)	0.36 ($\pm 0.01$)	0.65 ($\pm 0.01$)
VegaZINB	ZIPoisson	(llperm)	0.65 ($\pm 0.01$)	0.06 ($\pm 0.00$)	0.65 ($\pm 0.01$)	0.84 ($\pm 0.01$)
VegaZINB	NegativeBinomial	(loglik)	0.49 ($\pm 0.01$)	0.01 ($\pm 0.00$)	0.76 ($\pm 0.01$)	0.86 ($\pm 0.01$)
VegaZINB	NegativeBinomial	(llperm)	0.80 ($\pm 0.01$)	0.05 ($\pm 0.00$)	0.80 ($\pm 0.01$)	0.89 ($\pm 0.00$)
VegaZINB	ZINegativeBinomial	(loglik)	0.78 ($\pm 0.01$)	0.06 ($\pm 0.00$)	0.75 ($\pm 0.01$)	0.87 ($\pm 0.00$)
VegaZINB	ZINegativeBinomial	(llperm)	0.76 ($\pm 0.01$)	0.05 ($\pm 0.00$)	0.76 ($\pm 0.01$)	0.88 ($\pm 0.00$)

We see in Table 11 that parametric distributions based on likelihood, in particular the zero-inflated Negative Binomial, perform very well when the data set is in fact generated from a known parametric distribution (‘dmn_se’, ‘sparseDOSSA’, ‘metaSPARSim’, ‘VegaZINB’). With real data sets the situation can be different, depending on the data set. The real ‘soilrep’ data set seems to have considerably less zero-inflation and overdispersion because even the Poisson distribution works somewhat, and the standard Negative Binomial distributions seem satisfactory. The real ‘enterotype’ data set seems to behave much like our VEGA data set and we replicate our findings for the necessity of the permutation approach.

One surprising finding is that ‘llperm’ can have an FPR slightly over 0.05, even though in theory the permutation approach should guarantee the nominal level. We traced this back to the way signal is simulated in these kind of experiments: signal is added to data by increasing counts in 10% of the taxa in a particular group, which will increase the library size in this group. This will then automatically decrease the relative abundance of all other taxa, meaning that a very weak signal is generated for all other taxa even though their counts stay the same. In other words, if the data is interpreted as fractions like $p_{i,j}=\frac{Y_{i,j}}{\sum _{j}(Y_{i,j})}$, taking one *j* and increasing $Y_{i,j}$ will not only alter $p_{i,j}$ but also all other $p_{i,k}$ where $k \ne j$. This effect is almost neglible if the library size is very large relative to a given OTU count, but otherwise the ’not differentially abundant’ taxa can also be detected as changed. We verified that without the library size offset the method has exactly 0.05 FPRs. This implies that simulation methods could be further improved by adding signal in way that keeps the library size constant, that is by both adding and subtracting counts.
